# Extensive Natural Variation for Cellular Hydrogen Peroxide Release Is Genetically Controlled

**DOI:** 10.1371/journal.pone.0043566

**Published:** 2012-08-29

**Authors:** Homa Attar, Karen Bedard, Eugenia Migliavacca, Maryline Gagnebin, Yann Dupré, Patrick Descombes, Christelle Borel, Samuel Deutsch, Holger Prokisch, Thomas Meitinger, Divya Mehta, Erich Wichmann, Jean Maurice Delabar, Emmanouil T. Dermitzakis, Karl-Heinz Krause, Stylianos E. Antonarakis

**Affiliations:** 1 Department of Genetic Medicine and Development, University of Geneva Medical School, Geneva, Switzerland; 2 Department of Pathology and Immunology, University of Geneva Medical School and University Hospitals, Geneva, Switzerland; 3 Swiss Institute of Bioinformatics, University of Lausanne, Dorigny, Switzerland; 4 Genomics Platform, NCCR Frontiers in Genetics, University of Geneva Medical School, Geneva, Switzerland; 5 Institute of Epidemiology I, Helmholtz Zentrum München, German Research Center for Environmental Health, Neuherberg, Germany; 6 Institute of Medical Informatics, Biometry and Epidemiology, Chair of Epidemiology, Ludwig-Maximilians-Universität, Munich, Germany; 7 Klinikum Grosshadern, Munich, Germany; 8 Functional and Adaptive Biology, Université Paris Diderot-Paris7 and CNRS, Paris, France; South Texas Veterans Health Care System and University Health Science Center San Antonio, United States of America

## Abstract

Natural variation in DNA sequence contributes to individual differences in quantitative traits. While multiple studies have shown genetic control over gene expression variation, few additional cellular traits have been investigated. Here, we investigated the natural variation of NADPH oxidase-dependent hydrogen peroxide (H_2_O_2_ release), which is the joint effect of reactive oxygen species (ROS) production, superoxide metabolism and degradation, and is related to a number of human disorders. We assessed the normal variation of H_2_O_2_ release in lymphoblastoid cell lines (LCL) in a family-based 3-generation cohort (CEPH-HapMap), and in 3 population-based cohorts (KORA, GenCord, HapMap). Substantial individual variation was observed, 45% of which were associated with heritability in the CEPH-HapMap cohort. We identified 2 genome-wide significant loci of Hsa12 and Hsa15 in genome-wide linkage analysis. Next, we performed genome-wide association study (GWAS) for the combined KORA-GenCord cohorts (n = 279) using enhanced marker resolution by imputation (>1.4 million SNPs). We found 5 significant associations (p<5.00×10−8) and 54 suggestive associations (p<1.00×10−5), one of which confirmed the linked region on Hsa15. To replicate our findings, we performed GWAS using 58 HapMap individuals and ∼2.1 million SNPs. We identified 40 genome-wide significant and 302 suggestive SNPs, and confirmed genome signals on Hsa1, Hsa12, and Hsa15. Genetic loci within 900 kb from the known candidate gene p67phox on Hsa1 were identified in GWAS in both cohorts. We did not find replication of SNPs across all cohorts, but replication within the same genomic region. Finally, a highly significant decrease in H_2_O_2_ release was observed in Down Syndrome (DS) individuals (p<2.88×10−12). Taken together, our results show strong evidence of genetic control of H_2_O_2_ in LCL of healthy and DS cohorts and suggest that cellular phenotypes, which themselves are also complex, may be used as proxies for dissection of complex disorders.

## Introduction

Recent genome-wide association studies (GWAS) using clinical phenotypes have identified a large number of DNA polymorphisms that convey an increased risk for common diseases. Identified genetic loci, however, explain only a small fraction of the overall observed phenotypic variability even when assessing very large sample sizes [1]–[8] owing to biological complexity [9] and low penetrance of common genomic variants [10]. Small-scale studies (<100 individuals) indicate that gene expression is genetically controlled [11]–[13] emphasizing the direct link between phenotypic and genotypic variation. Despite these findings, how individual genes affect disease mechanisms remains poorly understood. Working with end-members of the phenotypic spectra (gene expression or clinical phenotypes) may not be sufficient to understand disease mechanisms. To remedy some of these shortcomings, we propose to explore the genetic control of cellular phenotypes (endophenotypes) as a trait of intermediate complexity. Our study will explore hydrogen peroxide (H_2_O_2_) release which is associated with multiple diseases as a proxy for a complex phenotype.

Hydrogen peroxide (H_2_O_2_) release is a quantitative cellular phenotype with known implications in multiple diseases [14]. H_2_O_2_ release, as measured at the extra-cellular level, includes reactive oxygen species (ROS) generation, and superoxide metabolism and degradation, all fundamental components of innate immunity. Causative associations between the phagocyte NADPH-dependent oxidase NOX complex and multiple diseases are known [14]–[17]. Both, low and high levels of ROS are detrimental and confer susceptibility to distinct diseases. Among these, low levels of ROS production have been shown to be associated with X-linked chronic granulomatous disease (CGD), autosomal recessive CGD, autoimmunity, and arthritis [18]–[23]. High ROS levels have been attributed to cardiovascular disease, hypertension, and neurodegenerative disorders [24]–[32]. The first evidence for genetic factors involved in ROS level regulation comes from a family-based study assessing heredity of ROS levels in essential hypertension [24]. This study found that 20–35% of the observed variation could be attributed to genetic factors. Down Syndrome (DS) individuals also show a ROS alteration suggesting that genetic loci on Homo sapiens (Hsa) chromosome 21, or loci on other chromosomes interacting with Hsa21, may be key players in ROS regulation. Despite efforts to characterize both the genetic and the molecular basis of altered ROS production in DS, insight into disease mechanisms are still lacking [33], [34]. Some of the phenotypes of DS might be related to unbalanced redox-states. However, the role, impact, and consequences of low or high ROS levels in various cell types of this chromosomal abnormality remain obscure [35]–[39].

The major source of ROS production in lymphoblastoid cells is NOX2-dependent. NOX2-deficient lymphocytes from chronic granulomatous disease (CGD) patients produce very low levels of ROS. Expressed co-factors of NOX2 and NOX homologues in lymphoblastoid cell lines include p22^phox^, p47^phox^, p67^phox^, p40^phox^, RAC1, and RAC2. Intracellular superoxide produced by this enzyme can not diffuse through cell membranes. However, superoxide is readily converted both spontaneously and enzymatically, to hydrogen peroxide (H_2_O_2_), which is membrane permeable. Superoxide dismutase (SOD), catalase, and peroxidases are associated with ROS metabolism and degradation. In this study, we measure ROS production and H_2_O_2_ release by detecting H_2_O_2_ release at the extra-cellular level.

The aim of this study is to evaluate the use of a cellular quantitative phenotype as a proxy to complex phenotypes by taking H_2_O_2_ release in EBV transformed lymphoblastoid cell lines (LCL) as an example. We aim at identifying human genetic factors underlying the variability of H_2_O_2_ release, and thus the susceptibility to infectious diseases, through a whole genome linkage and association analysis in the general healthy population. Furthermore, we evaluated levels and differences of H_2_O_2_ release in LCL derived from Down Syndrome individuals, and identify regulatory loci which may provide insights into the biological pathways of H_2_O_2_ variation.

## Results

### Phenotypic distribution of H_2_O_2_ release in four population-based cohorts

**Figure 1:**
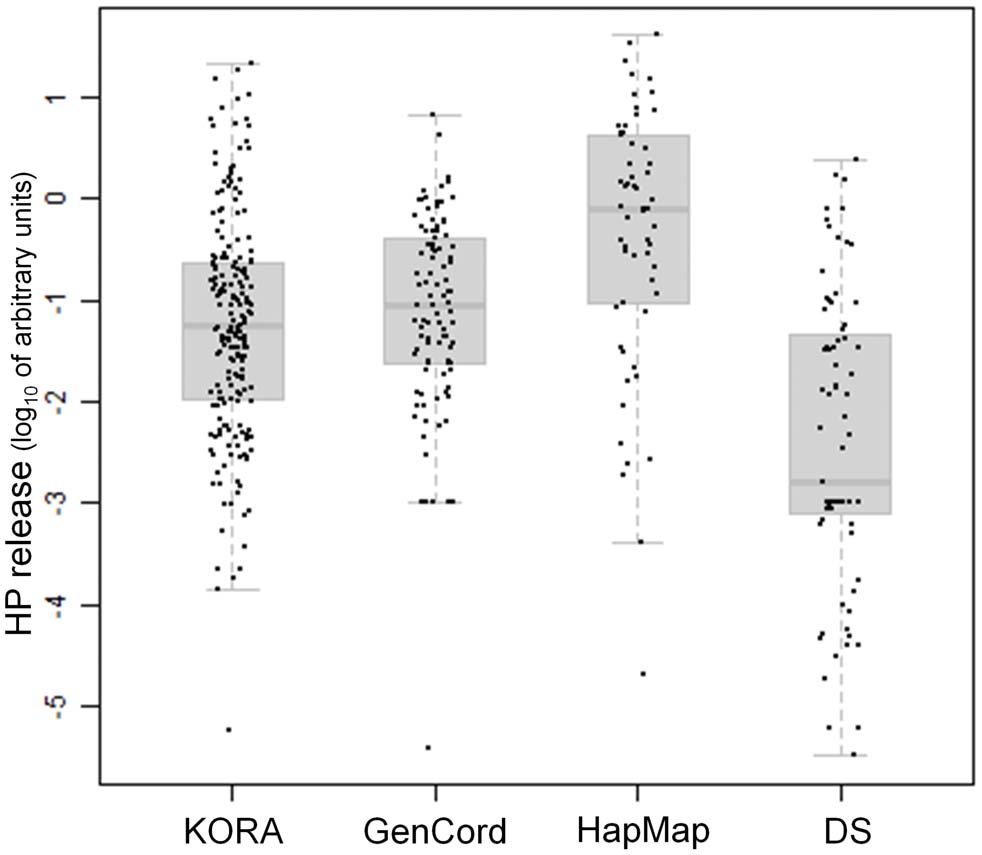
Distribution of H_2_O_2_ release in sample cohorts KORA, GenCord, HapMap and Down Syndrome. Box plots (in light grey) for the sample cohorts KORA, GenCord, HapMap and Down Syndrome (DS) show the distribution of H_2_O_2_ variation across cohorts. Each black dot corresponds to an individuals' mean of 4 measures of H_2_O_2_ release. The median is shown as a dark grey line, the low and upper end of the box plots (in light grey) indicate the first and third quartile of the distribution. The whiskers indicate the lower and upper end of the distribution The distribution of H_2_O_2_ in the Down Syndrome cohort shows a significant decrease as compared to HapMap (Wilcoxon rank sum test, p<2.88×10^−12^).

We observed extensive natural variation of NADPH oxidase-dependent H_2_O_2_ release in LCL of four population-based cohorts: KORA, GenCord, HapMap, and DS (**Figure 1**). KORA is a cohort of newly established LCL derived from unrelated healthy adults, GenCord is a cohort of newly established LCL derived from unrelated newborns, and HapMap is part of a three-generational sample cohort, from which unrelated LCL from healthy adults were used for genome wide association (GWA) analysis. A cohort of newly established LCL derived from unrelated Down Syndrome individuals were used to quantify the difference of these individuals to healthy cohorts. All cohorts are of Caucasian origin. A description of each cohort's demographic, phenotypic, and genotypic characteristics is shown in **Table 1**. We observed a mean and standard deviation for H_2_O_2_ release (arbitrary units) of 0.51 in the KORA cohort (±0.64, N = 199), 0.47 (±0.38, N = 80) in GenCord, 1.22 (±1.15, N = 58) in HapMap, and 0.23 (±0.31, N = 75) in DS individuals (**Figure 1** **,** **Table 1** **, File S1**). To quantify the degree to which this observed variation was individual-specific and reproducible, we assessed phenotypes in 21 LCL of unrelated HapMap individuals in two independent experiments. We found a 28-fold inter-individual difference, and high reproducibility (ratio of coefficient of variance >28, p<1.00×10^−4^, **File S2A**, Pearson correlation coefficient = 0.89, p<1.00×10^−4^, **File S2B**). The phenotypic distribution of H_2_O_2_ release in the DS cohort differed from all other healthy cohorts, with a significantly different distribution compared to the HapMap (Wilcoxon rank sum test, p<2.88×10^−12^), the GenCord (p<3.11×10^−9^), and the KORA cohort (p<5.35×10^−9^, **Figure 1**).

**Table 1:** Demographic characteristics for all four population-based cohorts.

	GENCORD	KORA	CEPH-HapMap	Down Syndrome
**Cohort characteristics**				
Origin cohort	University of Geneva, Laboratory of Prof. Antonarakis SE, Geneva, Switzerland	German Research Center for Environmental Health, Neuherberg, Germany	Coriell Cell Repositories, Camden, USA	AnEUploidy collection, Paris, France; Galliera Genetic Bank of Genoa, Italy; Emory University School of Medicine, Atlanta, USA and CSS-Mendel Institute in Rome, Italy
Origin population	Caucasian	Caucasian	Caucasian	Caucasian
Sample size	99	200	58	75
Individual's age (category)	1 day	40–60 years	>60 years	<40
Immune status	immature	mature	mature	mixed
Recently established cell line	yes	yes	no	yes
Gender (% female)	49%	56%	52%	-
**Phenotypic characteristics (H_2_O_2_ release)**				
Mean	0.47	0.51	1.22	0.22
Standard deviation	±0.38	±0.64	±1.15	±0.31
Range	0.004–2.28	0.005–3.73	0.009–4.97	0.004–1.45
**Genotypic characteristics**				
Plateform	Illumina 550 K	Affymetrix 6.0 500 K	multiple plateforms	no genotypes
Number called SNPs	480,455	413,877	2.1 million	-
Number called and imputed SNPs	1.4 million	1.4 million	2.1 million	-

Demographic, phenotypic and genotypic characteristics for each cohort are indicated.

We next aimed at identifying the genetic contribution to this natural variation in H_2_O_2_ release. Linkage analysis was used as a first step to assess the genetic basis of H_2_O_2_ release in the family-based HapMap-CEPH cohort, and genome-wide association analysis was conducted to refine and replicate results in the KORA, GenCord, and HapMap cohorts.

### Hydrogen peroxide release heritability and linkage analysis

**Figure 2:**
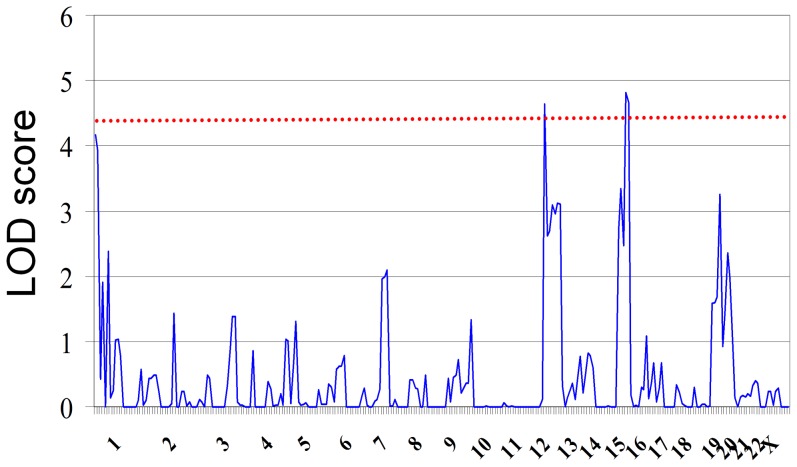
Two significant and two suggestive LOD scores on Hsa12, Hsa15, Hsa1, and Hsa19. LOD scores of genome-wide linkage analyses using the CEPH-HapMap family-cohort of three generations are plotted for all autosomal chromosomes (in blue). The blue line shows the maximal LOD score within a window size of 10 kb. The red dotted line indicates the significance level identified through 1'000 simulations.

We measured H_2_O_2_ variation in human LCL from 10 CEPH-HapMap three-generation families (N = 113). We observed extensive variation in H_2_O_2_ release that ranged from 0.001 to 6.035 (arbitrary units). To estimate what fraction of this natural phenotypic variation could be attributed to genetic components, we performed a variance-component linkage analysis [40] and found genome-wide estimated sample heritability (h^2^) of 45%. This finding suggests that 45% of all H_2_O_2_ release variation can be attributed to genetic factors in the cell lines used herein. In a linkage analysis of LCL of these same individuals, we identified two significant and two suggestive LOD scores on chromosomes Hsa1, Hsa12, Hsa15 and Hsa19 (**Figure 2**) with the use of 2723 SNP markers. Nominal genome-wide significance level was assessed by performing 1000 simulations in which we shuffled the phenotypes but kept the genotypes, allele frequency, and family structure constant. A LOD score of 4.48 was obtained for the nominal genome-wide significance threshold. The significant linkage signals were on Hsa12 with LOD score of 4.64 (p<1.00×10^−6^ for marker TSC0795922 at position 10,950 kb) and on Hsa15 with LOD score 4.81 (p<1.00×10^−6^ for marker TSC0181040 at position 37,068 kb). However, marker density in this linkage analysis was not sufficient to assign candidate genes. Furthermore, the linked region on Hsa12 is very gene-rich. The identified locus on Hsa15 is located 115 kb upstream of the transcription start site of MEIS2. The suggestive hit on Hsa1 had a LOD score of 4.17 (p<1.00×10^−5^, TSC0343078 at position 4,389 kb), the second suggestive linkage signal was identified on Hsa19 with a LOD score of 3.26 (p<5.00×10^−5^, TSC1058872 at position 54,754 kb).

### Genome-wide association analysis for KORA-GenCord

We used the KORA and GenCord cohorts as an initial combined cohort for GWA analysis and we used HapMap (N = 58) as a replication cohort (see discussion). As all three cohorts were genotyped on different platforms, we obtained a common and larger set of SNPs by imputation using the HapMap Phase II genotypes as a reference (see methods, **File S3**). After applying quality filters for imputed SNPs (R>0.9), a total set of 1.4 out of 2.1 million SNPs was included for GWAS. We performed principal component analysis to detect outliers for each cohort separately, and excluded one sample from the GenCord cohort. Next, we assessed whether the use of multiple cohorts and imputation of SNPs did not lead to population substructure, or clustering of called versus imputed SNPs by performing both principal component analysis and multidimensional scaling for the combined three cohorts. For the PCA analysis, a random selection of 500 K SNPs, and a centered approach was used. No evident substructure was detected by visual inspection among the three cohorts (**File S4**), and by analysis of proportions of variance of components 1 to 18. The cumulative proportion of variance of the first 10 components explained only 0.039 of the observed variation (data not shown), and increased slowly with increasing components.

**Figure 3:**
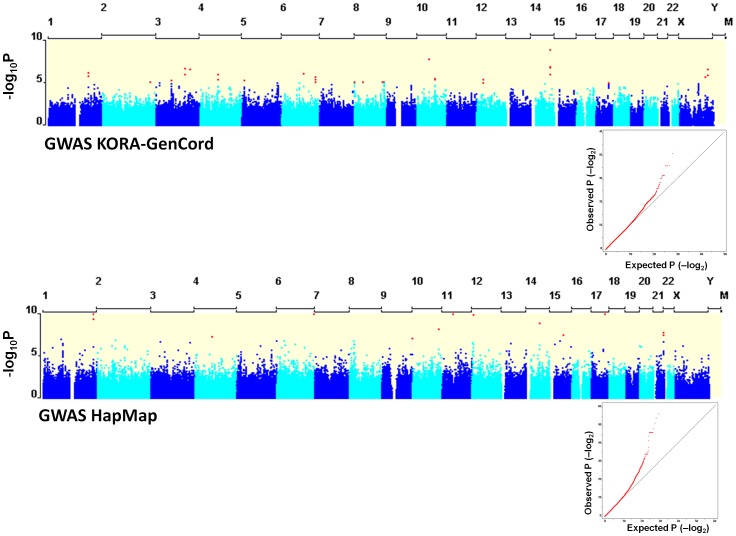
Genome-wide association results for natural variation of H_2_O_2_ release in KORA-GenCord and HapMap cohorts. Manhattan dot plots show results for genome-wide association analysis for the KORA-GenCord and the HapMap sample cohorts. The blue dots represent the association finding of one single SNP with natural variation in H_2_O_2_ release. Light and dark blue colors distinguish subsequent chromosomes. We did not analyze genetic markers for mitochondrial SNPs in this study. We show results of a genetic additive model indicating the unadjusted nominal −log_10_ of p-values. Quantile plots show the observed versus the expected distribution of GWAS for both analyses.

**Figure 4:**
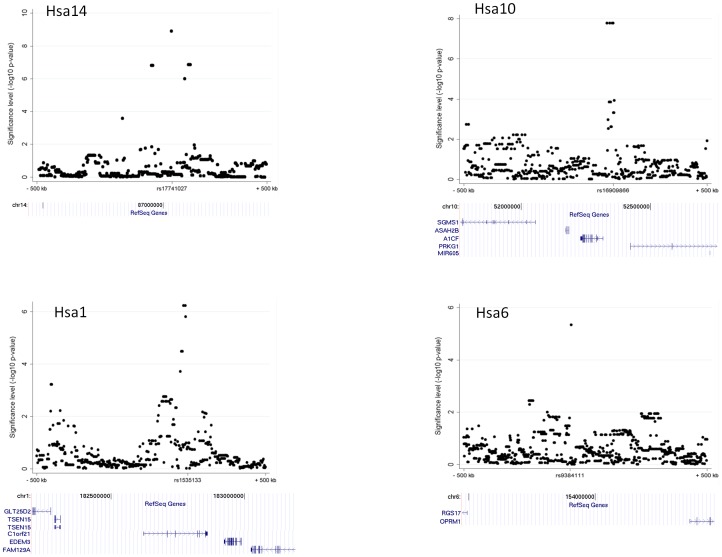
Examples of significant association findings in KORA-GenCord GWA. The significance level (−log_10_ P) of the top two association findings and two examples of top hits overlapping with either linkage, top GWA in HapMap, overlap with known candidate genes, or top findings on Hsa21 are shown within a genetic region of 1 Mb (±500 kb around the significantly associated SNP). The chromosome, SNP ID, location and genic context (RefSeq genes, using UCSC genome browser) are indicated for each depicted SNP.

We found 5 significant associations (p<5×10^−8^) and 54 suggestive associations with p-values of less than 1.00×10^−5^ (**Figure 3** **,** **Figure 4**) with the use of a linear genetic association model and median centered phenotypic distribution (see discussion for the choice of the design and the genetic model). These associated loci are located in 21 genetic clusters when defining a cluster as 1 mega-base of size (**Table 2** shows the top 15 clusters). Nominal unadjusted p-values from the WALD test statistics are reported. The strongest association hit was found on Hsa14 with a cluster of 7 SNPs on position 86,942 kb (top signal rs17741027, p = 1.23×10^−9^), followed by Hsa10 with 4 SNPs at position 52,264 kb (rs16909866, p = 1.66×10^−8^). Among these top associations (p<1.00×10^−5^), we could not find association on Hsa12 and Hsa15 within the genetic regions linked in the CEU-CEPH family-based cohort, but we found an overlap with a known candidate gene, p67^phox^ (also called NOXA2 or NCF2) on Hsa1. Three SNPs are located 913 kb downstream of the candidate gene p67phox on Hsa1 (position 181,791 kb), with the strongest signal for SNP rs1535133 (p = 5.97×10^−7^, position 182,704 kb (**Figure 4**). To replicate these findings, and to investigate whether the previously linked regions were population-specific, we subsequently analyzed the population-based HapMap cohort in a GWAS.

**Table 2:** Results from GWAS for the KORA-GenCord cohort.

SNP ID	Chr	Pos (bp)	type	Closest gene (within 1 MB)	Unadjusted p-value	Number SNPs/cluster
rs17741027	14	86941588	INTERGENIC	GALC	1.23×10−9	7
rs16909866	10	52263776	INTRONIC	ACF_HUMAN	1.66×10−8	4
rs1400014	3	132414349	INTRONIC	NEK11	2.27×10−7	2
rs3859991	X	130050216	INTRONIC	RHGXX_HUMAN	2.64×10−7	3
rs6801020	3	153876452	INTERGENIC	P2RY1	2.68×10−7	1
rs1535133	1	182704057	INTRONIC	C1orf21	5.97×10−7	3
rs9372321	6	97600416	INTRONIC	KLHL32	8.6×10−7	1
rs11728249	4	83760349	INTERGENIC	C4orf11	1.7×10−6	2
rs5909759	X	116093601	INTERGENIC	U6	2.13×10−6	3
rs9479282	6	152642425	INTRONIC	SYNE1;C6orf98	2.23×10−6	7
rs10887721	10	82035334	INTRONIC	MAT1A	3.17×10−6	2
rs4930939	12	30893752	INTERGENIC	CAPRIN2	4.58×10−6	3
rs9384111	6	153924030	INTERGENIC	U6	4.59×10−6	1
rs4701883	5	10816656	UPSTREAM	DAP	5.43×10−6	1
rs17006293	3	69777596	INTERGENIC	MITF	5.88×10−6	3

We show the results of the top 15 out of the 21 clusters of significantly associated genetic loci (p<5.00×10^−8^) and with suggestive associations (p<1.00×10^−5^) from genome-wide association analyses. We associated natural variation in H_2_O_2_ release measured in the KORA-GenCord cohort (n = 279 individuals) with more than 1.4 million markers (called and imputed SNPs). SNP ID, chromosome and position are indicated. Also, the type of SNP, the closest gene (within 1 MB), unadjusted p-value and the number of SNPs per cluster are indicated. SNP annotations were done by using the software WGAViewer (build 36, Ensembl core database *homo sapiens core 46 36h*, Ensembl variation database *homo sapiens variation 46 36h*, and Ensembl gene ontology database *ensemble go 46*).

### Genome-wide association analysis for HapMap

**Figure 5:**
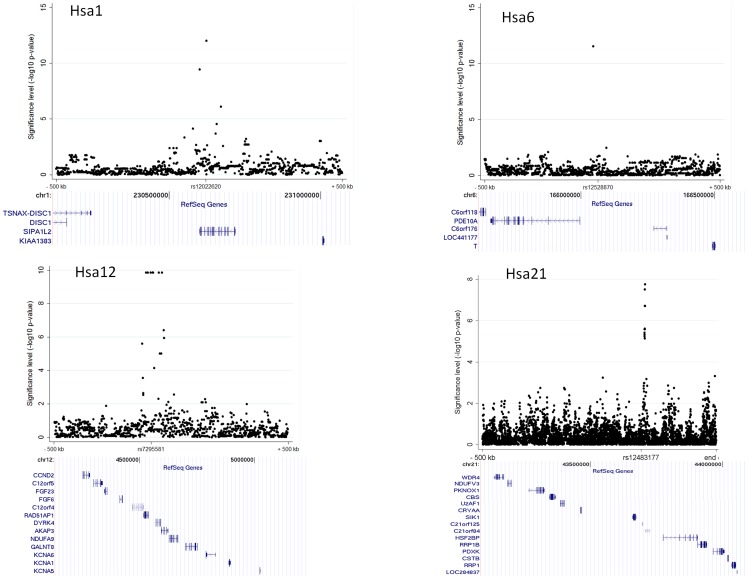
Examples of significant association findings in HapMap GWA. The significance level (−log_10_ P) of the top two association findings and two examples of top hits overlapping with either linkage, top GWA in KORA-GenCord, overlap with known candidate genes, or top findings on Hsa21 are shown within a genetic region of 1 Mb ((±500 kb around the significantly associated SNP). The chromosome, SNP ID, location and genic context (RefSeq genes, using UCSC genome browser) are indicated for each depicted SNP.

We used 60 unrelated LCL of Caucasian origin from the CEPH-Hapmap cohort as a replication cohort. We determined the H_2_O_2_ release in 58 samples and performed a GWAS using a total of 2,124,999 SNPs with minor allele frequency of more than 5% [41], [42]. We detected highly significant association signals in this analysis (**Figure 3** **,** **Figure 5**). Overall, 40 SNPs reached genome-wide significance of p<5.00×10^−8^, and 302 suggestive SNPs of less than p<1.00×10^−5^ that correspond to 90 clusters (**Table 3** shows the top 15 clusters). The most significant loci with p-values of less than 1.00×10^−12^ were rs12022620 on Hsa1 (intronic SNP within *SIPA1L2*) and rs12528870 on Hsa6 (intergenic SNP, **Figure 5**). The function of *SIPA1L2* (signal-induced proliferation-associated 1 like) is not well characterized yet. Among the other strong statistical signals, we identified association signals within the genomic regions of the linked regions. We found a cluster on Hsa12 (p<1.4×10^−10^) with 12 SNPs at position 4,690 kb close to *KCNA6* and *GALNT8*, and on Hsa21 (rs12483177, p<1.8×10^−8^) with 11 SNPs at position at 43,587 kb close to *SIK1* and *HSF2BP* (**Figure 5**). *KCNA6* (voltage-gated potassium channel) mediates the voltage-dependent potassium ion permeability of excitable membranes (UniProtKB for KCNA6_HUMAN). The role of *GALNT8* (polypeptide N-acetylgalactosaminyltransferase 8) is not described in the literature. *SIK1* (salt-inducible kinase 1) has a transient role during the earliest stages of myocardial cell differentiation and/or primitive chamber formation [43] and may also be important for the earliest stages of skeletal muscle growth and/or differentiation and in G2/M cell cycle regulation [44]. In computational studies investigating Hidden Markov Chain predictions and fold recognition algorithms for kinases on HSA21, *SIK1* and three other kinases were identified to be members of a novel mitogen-activated kinase pathway (MAPK) [45].

**Table 3:** Results from GWAS for the HapMap cohort.

SNP ID	Chr	Pos (bp)	type	Closest gene (within 1 MB)	Unadjusted p-value	Number SNPs/cluster
rs12022620	1	230616732	INTRONIC	SIPA1L2	1.00×10−12	3
rs12528870	6	166117266	INTERGENIC	U6	3.00×10−12	1
rs12602262	17	62290605	INTERGENIC	CACNG5	1.20×10−11	1
rs11039359	11	47704385	INTRONIC	FNBP4	4.80×10−11	1
rs7295581	12	4690362	INTERGENIC	GALNT8	1.45×10−10	12
rs17833323	14	58415000	INTERGENIC	DACT1	1.32×10−9	32
rs10749212	10	118166653	INTERGENIC	PNLIPRP3	7.27×10−9	7
rs12483177	21	43586879	INTERGENIC	Q6ZN03_HUMAN	1.75×10−8	13
rs6494367	15	61002704	INTERGENIC	TLN2	3.6×10−8	1
rs17001416	4	77250823	INTRONIC	ART3	4.84×10−8	20
rs2297721	9	138776337	INTRONIC	NP_976222.1	8.64×10−8	3
rs17005290	2	81322921	INTERGENIC	5S_rRNA	1.45×10−7	3
rs466886	6	33348677	INTRONIC	RPS18	1.67×10−7	1
rs16947598	15	43815915	INTERGENIC	SQRDL	1.70×10−7	1
rs333036	8	19026645	INTERGENIC	PSD3	1.76×10−7	11

Results show the top 15 out of the 90 clusters of significantly associated genetic loci (p<5.00×10^−8^) and with suggestive associations (p<1.00×10^−5^) from genome-wide association analyses. We associated natural variation in H_2_O_2_ release measured in the HapMap cohort (n = 58 individuals) with more than 2.2 million markers. SNP ID, chromosome and position are indicated. Also, the type of SNP, the closest gene (within 1 MB), the unadjusted p-value and the number of SNP per cluster are indicated. SNP annotations were done by using the software WGAViewer (build 36, Ensembl core database *homo sapiens core 46 36h*, Ensembl variation database *homo sapiens variation 46 36h*, and Ensembl gene ontology database *ensemble go 46*).

### Comparisons across all linkage, association studies and known candidate genes

We identified replication between the top linkage findings for four loci in the HapMap samples on Hsa1 (rs2500247 at position 3,172 kb, p = 4.65×10^−6^), Hsa12 (rs10492058, 4,756 kb, p = 1.45×10^−10^), and on two loci on Hsa15 (rs2075814, 22,717 kb, p = 4.2×10^−7^; rs16947598, 43,816 kb, p = 1.70×10^−7^). Of the known candidate genes expressed in LCL, p67^phox^ (Hsa1, pos = 183,525 kb) overlapped with two top association signals in the KORA-GenCord GWAS and in the HapMap GWAS (KORA-GenCord, rs10489726, 913 kb downstream, p = 5.97×10^−7^; and HapMap, rs528846, 932 kb upstream, p = 8.63×10^−6^). We did not detect strong association findings in KORA-GenCord samples that overlapped with the linkage findings. Neither did we find the same replicated SNPs nor genetic regions among the two GWA studies within a distance of 1 Mb. **Table 4** indicates replicated loci among linkage and GWA.

**Table 4:** Overlap between top hits from linkage and GWAS.

Results for linkage and overlap with GWAS	Hsa1	Hsa12	Hsa15	Hsa19
**Linkage results (LOD>3)**	**TSC0774051 - TSC0393286**	**TSC0795922 - TSC0431222**	**TSC0453546 - TSC1202583**	**TSC1058872 -TSC0057999**
	1,864 kb–7'905 kb	10,841–13'493 kb	21,775–21'897 kb	59,446–59,469 kb
	LOD = 4.17	LOD = 4.64	LOD = 3.34	LOD = 3.26
		**TSC0796032-TSC0014204**	**TSC0181040-TSC0940969**	
		54,428–54,474 kb	34,855–37,771 kb	
		LOD = 3.09	LOD = 4.81	
		**TSC0209972-TSC0621539**	**TSC0577078**	
		96,905–99,714 kb	43,404 kb	
		LOD = 3.12	LOD = 4.4	
**KORA-GenCord (top associations) overlap with linkage**	-	-	-	-
**HapMap (top associations) overlap with linkage**	**rs2500247**	**rs10492058**	**rs2075814**	-
	3,172 kb	4,756 kb	22,717 kb	
	p = 4.65×10^−6^	p = 1.45×10^−10^	p = 4.2×10^−7^	
			**rs16947598**	
			43,816 kb	
			p = 1.70×10^−7^	

This table indicates replicated genetic regions identified through GWA in KORA-GenCord and in HapMap. Top hits for GWAS are shown when the loci overlap within 5 Mb of distance from top linkage hits. The chromosome, the genetic marker, the position and the LOD score or the p-value is shown.

Given the lack of replication of the top SNPs across the two GWA studies, we next asked whether we could detect enrichment of lower p-values of these top SNPs of the KORA-GenCord in the GWA HapMap. We observed an enrichment for p-values of p<0.2 in HapMap GWAS (data not shown). A similar pattern was observed when comparing top HapMap signals with p-values of all KORA-GenCord. This pattern suggests enrichment for repeated associated loci across GWAS, but this enrichment is not significant.

### Covariate analysis

To assess the impact of potential co-variates (immune status, time of cell line establishment, individuals' age, and gender) on H_2_O_2_ distribution, we combined all samples after median centered normalization, and estimated the relative contribution of each of the co-variates in an univariate linear regression model with a cluster effect on the population. We found that none of the covariates had a significant effect on the trait distribution. We found the following relative order of correlation: Time of establishment of cell lines (B-coefficient = 0.142, p = 0.231), status of immune system ((B-coefficient = 0.139, p = 0.127), individuals' age (B-coefficient = 0.109, p = 0.072), and gender (B-coefficient = 0.052, p = 0.499). To estimate whether a statistical model with adjustment for all co-variate was different than a model for the main variate only, we performed a randomized set of 100'000 SNPs using a multivariate linear regression model with adjustment for all co-variates and a cluster effect on the population. We compared the results of this model with an univariate model with no adjustment for co-variates. We found no significant differences between the two models for the 100'000 randomly selected SNPs. This analysis suggested that covariates did not represent a confounding factor.

## Discussion

The most essential outcome of this study is the finding of extensive natural phenotypic variation in healthy individuals for H_2_O_2_ release, and evidence for a genetic basis to the observed variation in LCL of the cohorts used herein. This finding is in line with several studies reporting large natural phenotypic variation, such as for gene expression level variation, many of which were repeatedly shown to be shaped by genetic polymorphisms [46]–[50]. It may be conceivable that many traits maintain natural variation for response to selection pressure [51]. The measurements of the phenotypic spectra of H_2_O_2_ release in more than 500 individuals in 4 healthy cohorts and in a DS cohort confirm previously reported differences in H_2_O_2_ release of primary neutrophils of healthy individuals and individuals with chronic granulomatous disease [52], and on altered redox-states in DS individuals.

In the study of the Kuhns DB, et al., 10–20 fold differences in primary neutrophils from peripheral blood of healthy individuals were detected in comparison to individuals with chronic granulomatous disease. Also, large inter-individual differences were detected, and correlated to genotypes. This correlation was reported to be a strong predictor for overall survival of CGD patients. Our genetic study of natural variation in H2O2 release revealed 45% of heritability, and we detected genomic regions on Hsa1, Hsa12 and Hsa15 as significant and replicated regions in more than one cohort. Results from GWAS in HapMap samples indicated an associated genetic locus close to the gene *SIK1* on Hsa21. *SIK1* is encoding a kinase known for its involvement in cardiomyogenesis by regulating cardiomyoblast cell cycle [43], [44], but it could also exert additional unknown functions. Further experiments are necessary to replicate and to assess the functional consequences of this association in DS and in healthy individuals. In our study, we did not find significant association signals on Hsa21 or any other markers close to *SIK1* in the combined KORA-GenCord GWA.

H_2_O_2_ release, an endophenotype more closely related to sequence variation (as opposed to a clinical phenotype) may be a complex trait in itself. The detection of multiple genome-wide significant loci suggests a polygenic trait. The strongest linkage results indicate loci on Hsa1, Hsa12, Hsa15 and Hsa19, two of which (Hsa1 and Hsa15) were also significantly associated in either the KORA-GenCord or HapMap GWAS, or both. However, we did not detect the same associated SNPs in the three cohorts, but replication within the same genomic regions. This may indicate either a lack of power to detect genetic loci due to the relatively small sample size, or reflect the complex genetic architecture of H_2_O_2_ with multiple loci contributing small proportions to the observed phenotypic variation. Our study design focused on the detection of common alleles with a genetic effect of more than 5% given an allele frequency of 0.15, and with our study design, we have 80% of power to detect such a quantitative trait locus while using 300 individuals [53].

Our study design considered the use of a combined GWA of 279 individuals to increase sample size, and to replicate findings in a follow-up cohort of 58 HapMap individuals. Even though all cohorts used herein are of Caucasian origin, and population substructure did not appear to be a confounding factor, differences among these cohorts may have reduced the power to detect the same loci across cohorts. Co-variate analysis showed that the effect of the SNP on association was significant in shaping the H_2_O_2_ trait, and suggests that despite differences in the origin of cohorts, cell line establishment and age of the cell lines the underlying genetics is the predominant factor. Further evidence for a strong genetic effect, rather than differences in cellular phenotypes in different cohorts, comes from a study by Dimas, et al. [13] showing that 83% of detected eQTLs in HapMap were replicated in GenCord samples. This finding was crucial to our study design and validates the use of the GenCord cohort as a comparable cohort to the HapMap cohort. We cannot exclude that H_2_O_2_ release may underlie different cellular covariates than gene expression levels, leading to population-specific differences that we could not fully correct for. We investigated multiple genetic models for assessing the best method for normalization of the phenotypic distribution. We expected all cohorts to show similar distributions of H_2_O_2_ release, as all individuals were of European ancestry, and we measured all phenotypes under standardized and highly controlled conditions. No substantial differences among cohorts could be attributed to population substructure or to known covariates. The non-normal distribution of phenotypes may probably be due to the relative small sample size, as departure from normal distribution was largest for HapMap (N = 58) and smallest for KORA (N = 199). As shown in Figure 1, the distribution of H_2_O_2_ release is enriched for extreme phenotypes with individuals with either very high or very low phenotypes. To assess the impact of the phenotypic distribution on our results, and to exclude false positive signals due to departure from model assumptions, we have considered three different methods of normalization across cohorts. Also, we compared results from parametric and non-parametric association models. We conducted log transformation, median centered and inverse variance normalization, and median centered normalization only. Log transformation did not lead to a normal distribution. When using median and inverse variance normalization we lost all association signals, leading to the hypothesis that the genetic signal may be associated with the high and low end distribution of the phenotypes. Therefore, we chose to use the median centered approach, while keeping the internal variation of each population untouched. The median centered approach does not fully support the assumption for normal distribution. To assess whether results differed due to model assumptions, we compared these results with a non-parametric regression model using the Spearman rank correlation. We found the same top association signals with both models, with the only difference of slightly lower p-values (mostly at one order of magnitude for the top signals), showing that results did not depend on model assumptions.

LCL represent a relevant physiological cell type for ROS production [14], and LCL express mainly NOX2-dependent ROS [54]–[56]. A study by Kobayashi, et al. [57] compared fresh neutrophils, fresh B-lymphocytes, and EBV transformed B-lymphocytes from the same individuals to compare the relative expression of NOX2. They reported that B-lymphocytes have 1.1% of the NOX2 and 2.6% of the p22 that a neutrophil has, and that this is further decreased when the cells are EBV transformed. They showed that regardless of the amount of NOX2 the neutrophil expressed, the relative expression level in a B-cell of the same individual was correlated. This study would not have been feasible without the use of immortalized cell lines for both large families and population-based collections with more than 2 million genetic markers. As for all genome-wide studies using cell lines, it is a first step into identifying the genetic basis and requires validation in further cohorts, functional analysis, and ultimately verification in primary cells from healthy and diseased individuals. Potential confounding factors due to EBV-transformation remain a subject of debate and need to be considered. Also, we measured H_2_O_2_ levels after PMA stimulation, and genetic variation involved in the *protein kinase C*, through which PMA acts directly, cannot be fully excluded. However, heritability would not have been detected if EBV transformation or PMA stimulation would affect pathways in a random way. Finally, H_2_O_2_ release is an endophenotype with multiple levels of metabolism. Our assay relied on extracellular measures of hydrogen peroxide release which is the joint result of ROS production, metabolism, and degradation. Each step of these mechanisms may involve few or many genes, and thus contribute to the complexity of the phenotype.

We did not find genome-wide significant associations for loci of the NADPH-dependent NOX2 and other NOX related genes. We identified a locus 1 Mb from p67phox (or NCF2) as the closest hit for NOX2 members expressed in LCL (p22^phox^, p40^phox^, p47^phox^, p67^phox^, and homologues RAC1 and RAC2). These results suggest that variation in other genetic loci have stronger impact on natural variation of H_2_O_2_ as compared to the known genes identified in studies of Mendelian disorders. Previous case-control studies investigating the molecular basis of either H_2_O_2_ or ROS applied candidate gene approaches with contradictory results. Some studies identified mutations or polymorphisms associated with ROS generation within the NADPH-dependent NOX2 gene or its cofactors, while others found no association [31]. Recently, it has been suggested that haplotype rather than single SNP analysis may capture more of the relevant biological effects of phenotypic traits. Indeed, in the case of NOX2 dependent ROS generation, Bedard and coworkers [16] report an association between p22^phox^ (also called *CYBA*) haplotypes, rather than single polymorphisms. The associated haplotype was shown to act, at least in part, through an effect on protein expression levels. While this approach shed some light on the contradictory results reported so far on the involvement of NADPH-dependent NOX components in ROS generation, the large observed natural variation could not solely be attributed to the NADPH-dependent NOX2 and its cofactors, leading to the hypothesis that other loci are involved in regulatory mechanisms.

Taken together, our results indicate that there is a substantially large contribution of genetic factors at the basis of H_2_O_2_ release among healthy individuals in the cohorts used in this study. Our results suggest that this trait, even though an endophenotype, is complex and that multiple genes or regulatory elements are probably acting in concert to shape its expression. The herein identified association findings, together with the closeby genes may be enriched for biologically significant genes, and/or genetic loci relevant to pathogen defense and signaling, and pathway analysis may constitute the next step into the understanding of the regulation of H_2_O_2_ release. More generally, the elucidation of the genetic control of some heritable cellular traits may also be a complex problem that requires combined approaches and extensive sample sizes.

## Materials and Methods

### Sample collections and genotyping

**CEPH_HapMap sample collection:** We acquired EBV-transformed lymphoblastoid cell lines (beta-LCL) of 10 families (1333, 1334, 1340, 1341, 1345, 1346, 1347, 1362, 1408, 13292) and of 60 unrelated individuals of Caucasian origin from the Coriell Cell Repositories (Coriell Cell Services, Coriell Institute for Medical Research, Camden, NJ, USA; http://ccr.coriell.org). These are considered as healthy individuals and form part of the Caucasians of European Ancestry (CEPH) and the Haplotype Map (HapMap) Project [41], [42]. Genotypes of the 10 families were downloaded from the CEPH database [50]. A total of 2723 single nucleotide polymorphisms (SNPs) were used for subsequent linkage analyses. For the 60 unrelated individuals, we used 2'124'999 million SNPs from publicly available databases, and from HapMart, NCBI_Build36 (http://snp.cshl.org/linkage_maps; www.hapmap.org; [41], [42].

**KORA sample collection:** KORA S4 (Cooperative Health Research in the Augsburg Region) is a sample collection of a population-based project conducted from 1999–2001 for which beta-LCL of healthy adults from the Augsburg region in Germany were established [58]. Two hundred LCL from this collection were used; these samples were genotyped with the Affymetrix 500 K oligonucleotide array containing a total of 500,568 SNPs (Affymetrix 6.0 chips). Genotypes were determined using the software BRLMM version 1.4.0 with standard settings proposed by the manufacturer. A total of 413,877 SNPs passed quality control filters and were used for further analysis.

**GenCord sample collection:** The GenCord (Geneva Cord sample collection, University of Geneva, Medical School, Geneva, Switzerland) sample collection is a population-based collection of LCL derived from umbilical cords of 200 healthy newborns of Western European origin. The sample collection was approved by the institutional ethics committee, and all mothers provided their informed consent prior to sample collection [13]. There are no phenotypic data on these newborns. DNA samples were extracted from cord tissue LCL with the Puregene cell kit (Gentra-Qiagen, Venlo, The Netherlands). Genotyping was performed with the illumina 550 K SNP (Illumina, San Diego, CA) array following the instructions of the manufacturers. From a total of 555,352 SNPs, 480,455 SNPs passed quality filters and were used for subsequent analysis.

Filter settings for genotype quality required accuracy of sample call rates of more than 98% were (estimated based on 5 duplicated genotyping of samples). Any SNP with a call rate of less than 99% call rate was excluded, as well as all SNPs called less than in 1% of all samples. Separation of homozygote and heterozygote SNPs was verified and corrected if needed (cluster separation >0.3 was required). The range of excess of heterozygosity was verified and SNPs not falling into the range of −0.1 to −1, and 0.1 to 1 were excluded. MAF was set to >5%; to exclude samples with cryptic relatedness, IBD was set to >0.125; and deviation of Hardy Weinberg equilibrium was defined as <0.05. We verified the specified gender, and visualized a random set of 100 SNPs for quality checking.

**Down syndrome sample collection:** 33 Down syndrome (DS) LCL samples were acquired from the Galliera Genetic Bank of Genoa, Emory University School of Medicine in Atlanta, and CSS-Mendel Institute in Rome [59] and 42 LCL from the AnEUploidy collection in Paris, France. Full trisomy was assessed by karyotyping. We excluded potential clustering effects of H_2_O_2_ release due to the multiple origins of these samples, no substructure was detected.

**Cell culture:** The LCL of all three populations were cultured in RPMI 1640 with Glutamax I medium (Invitrogen) supplemented with 10% fetal calf serum and 1% mix of penicillin, fungizone, and streptomycin (Invitrogen). All cell lines were grown and maintained for at least 2 weeks at a constant density of 200–400,000 cells/ml. Cells were washed once with PBS (Invitrogen) to reduce accumulation of cell debris 4–5 days prior to measuring ROS production.

**AmplexRed ROS measurements:** Cell lines were assessed for H_2_O_2_ release using AmplexRed (Invitrogen), a quantitative enzymatic assay that detects H_2_O_2_ in the culture media. Ninety minutes prior to AmplexRed measurements, cell lines were centrifuged at 1000 rpm for 10 min and transferred to 2,5 ml Hanks' balanced salt solution (HBSS) media (containing 5% of glucose) and kept on ice. 0.5 ml of each cell line was used for measuring cell number by Fluorescent Activated Cell Sorting (FACS). Beads were added at a known concentration to obtain absolute values of cell number. Cells were diluted to a final concentration of 500,000 cells/ml.

AmplexRed measurements were performed according to manufacturers' instructions with minor modifications. In brief, 50,000 cells were plated in 0.2 ml volume of HBSS containing 25 mM AmplexRed and 0.005 U/ml horseradish peroxidase. NADPH oxidase activity was stimulated with 0.1 mM phorbol 12-myristate 13-acetate (PMA). In 1 of 5 replicates per individual cell line, 10 mM diphenyleneiodonium (DPI), an NADPH oxidase inhibitor [60], was added prior to stimulation. Fluorescence was recorded every minute for a minimum of 30 min with excitation and emission wavelengths of 550 nm and 600 nm, respectively in a BMG Fluostar microplate reader at 37°C.

**Experimental design and normalization of H_2_O_2_:** Each well-plate was set up with 12 individual cell lines. Each cell line was measured in 4 replicates in addition to one replicate that was inhibited with DPI. From the 4 replicates per sample, one was removed based on an outlier detection procedure, and the average of the remaining 3 replicates was used as the phenotypic read-out. We defined the phenotypic read-out as the maximal slope over 5 time points within 30 min of measurements. The standard curve of H_2_O_2_ was used to transform relative H_2_O_2_ measures to absolute values for each individual plate, thus allowing for comparison among different plates. Experiments for which regression of the standard curve of H_2_O_2_ did not reach R^2^ of 0.99 were excluded. For normalization across plates, the same 2 individuals (GM0755 and GM07341) were measured on every plate and served as a reference for normalization. As a negative control cell line, an LCL of a patient with chronic granulomatous disease (CGD) was tested to ensure specificity of the assay for NOX2-dependent H_2_O_2_ detection.

### Statistical analysis

**Linkage analysis and heritability estimate:** Heritability analysis was performed with MERLIN software [40]. We assessed 113 individuals and their corresponding ROS production from 10 CEPH-HapMap families. Genetic markers for 2723 SNPs from the CEPH database (http://snp.cshl.org/linkage_maps); [61] were used. Mendelian inconsistencies and unlikely genotypes were removed using PEDCHECK and PEDWIPE software [62], [40].

For genome-wide linkage analysis, the same phenotypes and genotypes were used for multipoint variance component analysis [40]. Empirical significance of the linkage results were obtained from 1000 simulations by randomly redistributing the phenotypes and recalculating the maximal linkage signal genome-wide. We extracted the lowest p-value from each simulation to obtain a null distribution. Observed and expected p-values were compared to assess the nominal genome-wide significance threshold. All simulations were performed using R on a cluster of 32 HP/Intel Itanium 2 based servers at the Vital-IT Center, Lausanne, Switzerland (http://www.vital-it.ch). Mapping positions of these markers correspond to NCBI36/hg18.

**Normalization of phenotypes across populations:** We applied a median centered transformation for the phenotypes before combining the data from KORA and GenCord populations. HapMap samples were used as a replication cohort and were analyzed separately; and also analyzed with a median centered transformation.

**Association studies:** Imputation of SNPs across all 3 populations. We imputed SNPs from unphased alleles for 337 individuals by using the BEAGLE 3.0.4 software [63]. The HapMap sample collection with 2.2 million SNPs was used as the reference to impute SNPs for the KORA and GenCord collections. All SNPs were mapped to the same strand as the reference and A/T, C/G SNPs were discarded, resulting in 456,122, and 311,949 SNPs for KORA and GenCord, respectively. We set a threshold for the inclusion of an imputed SNP at allelic R^2^>0.9 to ensure high quality imputation. Of all 2.2 million imputed SNPs, 1.4 million were retained for further analyses.

**Principal component analysis:** Principal component analysis was first performed for samples of each cohort, and we excluded one sample from the GenCord population after visual inspection of the first 10 components. Principal component analysis of a random set of 500,000 SNPs was then used to test for population substructure among all three cohorts. Visual inspection of the first to the 8^th^ component did not reveal evidence for substantial population substructures.

**Genome-wide association studies:** We performed linear regression analyses and an additive genetic model to perform association between H_2_O_2_ levels and each SNP using PLINK software [64]. SNPs were coded 0, 1, or 2 for homozygous rare, heterozygous, and homozygous common alleles, respectively. For this additive model, we fitted a linear regression of the form
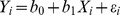
We report the nominal *p*-value of the test of no association, i.e., *b_1_ = 0* where Yi are the H_2_O_2_ release for each sample i, Xi are the genotypes coded as 0,1,2 and *ε_i_* are assumed to be independent normally distributed random errors with mean 0 and constant variance. Mapping positions of GWA markers for KORA/GenCord and for HapMap correspond to NCBI36/hg18.

**Covariate analysis:** We combined all samples from HapMap, KORA and GenCord after median transformation of the H_2_O_2_ release phenotype (N = 337), The following covariates were assigned to each individual: The status of the immune system of the individual (“mature” for HapMap and KORA, “immature” for GenCord); gender; the time of cell lines establishment prior to use in this study (“old” for HapMap, “new” for KORA and GenCord); the age of the individual (“>60 years” for HapMap, “40–60 years” for KORA, “1 day” GenCord). A clustering effect was set for each of the three populations. We conducted a general linear mixed effect regression model with a clustering effect for the population and adjustment for immune status, gender, cell line age, and individual age. We performed univariate mixed effect linear regression with a cluster effect on populations for each co-variate. We then used a multivariate mixed effect linear regression with adjustment for all co-variates and a cluster effect on populations for a randomized set of 100'000 SNPs. This model was compared to a univariate mixed effect linear regression using the SNP only.

## Supporting Information

File S1 **Sample collection identifiers and phenotypes.** All individuals used in this study are indicated with their identifier and their phenotype. H2O2 release indicates normalized hydrogen peroxide release levels, units are arbitrary.
(DOC)

File S2 **Extensive inter-individual variation and high reproducibility for H_2_O_2_ phenotypes.** (**A**) H_2_O_2_ release for 21 CEPH-HapMap unrelated individuals, each of which was measured in 4 replicates is shown. Each bar corresponds to the normalized H_2_O_2_ release of an individual, and standard deviations of three replicates per individual are indicated in red. The coefficient of variance is >28 with a p-value of p<1.00×10^−4^, indicating that there is 28 times more variation among individuals than among replicates. (**B**) H_2_O_2_ release for the same 21 individuals was measured several weeks later, to estimate the reproducibility of the phenotype. Comparison for individuals of both experimental sets indicates a coefficient of correlation of more than 0.89 (Pearson correlation test, p<1.00×10^−4^).
(TIF)

File S3 **Overlap of SNPs among the three sample cohorts and genotyping platforms.** This schematic figure displays the number of individuals genotyped for each cohort, and the number of SNPs genotyped per population. The overlap between the circles shows the numbers and percentages of overlapping SNPs among populations and genotyping platforms.
(TIF)

File S4 **Principal component analysis indicates no apparent substructure.** A random set of 500,000 SNPs of 337 individuals from three cohorts was analyzed using centered principal component analysis (PCA). Here we show PC1 versus PC2. No evident substructure is apparent among cohorts.
(PNG)
