# Supplementary material for: Extensive Natural Variation for Cellular Hydrogen Peroxide Release Is Genetically Controlled
Source: PLoS One. 2012 Aug 29;7(8):e43566. doi: 10.1371/journal.pone.0043566 (PMC3430705; doi:10.1371/journal.pone.0043566)
Supplement: File S1 — Sample collection identifiers and phenotypes. All individuals used in this study are indicated with their identifier and their phenotype. H2O2 release indicates normalized hydrogen peroxide release levels, units are arbitrary. (DOC) [file pone.0043566.s001.doc]

| **HapMap ID** | **H_2_O_2_ release** | **GenCord ID** | **H_2_O_2_ release** | **KORA ID** | **H_2_O_2_ release** | **DS ID** | **H_2_O_2_ release** |
| --- | --- | --- | --- | --- | --- | --- | --- |
| NA06985 | 0.7575 | UC007 | 0.0044 | 1780055 | 1.5566 | DS_001 | 0.1450 |
| NA06993 | 0.6553 | UC010 | 0.7231 | 1780124 | 0.5705 | DS_002 | 1.2430 |
| NA06994 | 0.9266 | UC012 | 0.7926 | 1780156 | 0.2728 | DS_003 | 0.6390 |
| NA07000 | 0.5859 | UC016 | 0.1283 | 1780375 | 0.1380 | DS_004 | 0.2440 |
| NA07022 | 0.1719 | UC018 | 0.9221 | 1780476 | 0.8790 | DS_005 | 0.2840 |
| NA07034 | 0.6364 | UC019 | 2.2844 | 1780738 | 0.2657 | DS_006 | 0.9000 |
| NA07055 | 0.9000 | UC022 | 0.1420 | 1780749 | 0.2207 | DS_007 | 0.1430 |
| NA07056 | 0.6219 | UC023 | 0.3814 | 1780828 | 0.3471 | DS_008 | 0.3550 |
| NA07345 | 0.3870 | UC024 | 0.8014 | 1780929 | 0.2084 | DS_009 | 0.8130 |
| NA07357 | 0.2197 | UC025 | 0.4406 | 1781241 | 0.3168 | DS_010 | 0.3880 |
| NA11829 | 0.1652 | UC026 | 0.5017 | 1781256 | 0.3823 | DS_011 | 0.3540 |
| NA11830 | 1.2769 | UC027 | 0.7288 | 1781400 | 0.0864 | DS_012 | 0.0500 |
| NA11831 | 1.8903 | UC028 | 0.6145 | 1781573 | 1.0794 | DS_015 | 0.8950 |
| NA11832 | 0.1296 | UC029 | 0.1444 | 1781683 | 0.0439 | DS_188 | 0.0500 |
| NA11839 | 1.4093 | UC032 | 0.1056 | 1781771 | 0.1532 | DS_189 | 0.0500 |
| NA11840 | 1.1000 | UC033 | 0.2725 | 1781864 | 0.1060 | DS_193 | 1.1840 |
| NA11881 | 4.9717 | UC034 | 0.3936 | 1781870 | 0.1482 | DS_194 | 0.6250 |
| NA11882 | 0.5626 | UC037 | 0.0500 | 1782011 | 0.6819 | DS_195 | 0.0400 |
| NA11992 | 0.8999 | UC039 | 1.2142 | 1782121 | 0.0052 | DS_198 | 0.0500 |
| NA11993 | 1.1667 | UC040 | 0.0500 | 1782176 | 1.6449 | DS_213 | 0.0500 |
| NA11994 | 2.8535 | UC041 | 0.2922 | 1782378 | 0.2612 | DS_219 | 0.0500 |
| NA11995 | 1.8953 | UC042 | 0.0500 | 1782412 | 0.1720 | DS_220 | 0.0500 |
| NA12003 | 0.5728 | UC043 | 0.1290 | 1782539 | 0.2510 | DS_221 | 0.1580 |
| NA12004 | 0.6663 | UC044 | 0.9633 | 1782591 | 0.4438 | DS_223 | 0.0480 |
| NA12005 | 0.0890 | UC045 | 1.0649 | 1782635 | 2.7909 | DS_234 | 0.0500 |
| NA12006 | 2.0439 | UC048 | 0.6100 | 1782747 | 2.1486 | DS_236 | 0.0500 |
| NA12043 | 1.6416 | UC049 | 0.5302 | 1782772 | 3.2636 | DS_237 | 0.0500 |
| NA12056 | 4.6064 | UC050 | 0.1836 | 1782837 | 2.0479 | DS_244 | 0.0500 |
| NA12057 | 3.3384 | UC051 | 0.1527 | 1782949 | 0.3565 | DS_253 | 0.0470 |
| NA12144 | 1.1179 | UC051 | 0.2133 | 1783012 | 0.0708 | DS_268 | 0.0500 |
| NA12145 | 2.7679 | UC055 | 0.3184 | 1783085 | 0.0322 | DS_279 | 0.0500 |
| NA12146 | 0.5080 | UC056 | 0.2526 | 1783122 | 0.5277 | DS_283 | 0.0500 |
| NA12154 | 0.8796 | UC057 | 0.1949 | 1783243 | 0.1299 | DS_290 | 0.0500 |
| NA12155 | 2.2885 | UC058 | 0.4233 | 1783366 | 0.3858 | DS_1 | 0.0231 |
| NA12156 | 0.3261 | UC060 | 0.6826 | 1783505 | 0.1709 | DS_2 | 0.2728 |
| NA12234 | 1.2174 | UC062 | 0.1167 | 1783564 | 0.0213 | DS_3 | 0.0469 |
| NA12236 | 0.0092 | UC063 | 0.1962 | 1783652 | 0.1021 | DS_4 | 0.1923 |
| NA12239 | 0.8275 | UC065 | 0.2554 | 1783762 | 0.0976 | DS_5 | 0.0134 |
| NA12248 | 1.1569 | UC068 | 0.0500 | 1783773 | 2.0099 | DS_6 | 0.0976 |
| NA12249 | 0.2309 | UC069 | 0.2574 | 1783872 | 0.5134 | DS_7 | 0.1144 |
| NA12264 | 0.0763 | UC070 | 0.2242 | 1783894 | 0.2116 | DS_8 | 0.0121 |
| NA12716 | 2.3755 | UC076 | 1.1287 | 1783948 | 0.1324 | DS_9 | 0.0121 |
| NA12750 | 0.0333 | UC078 | 0.3993 | 1783982 | 0.2343 | DS_10 | 0.0136 |
| NA12751 | 1.4098 | UC079 | 0.1487 | 1784168 | 0.4515 | DS_11 | 0.0054 |
| NA12760 | 2.4025 | UC081 | 0.1770 | 1784275 | 0.2544 | DS_12 | 0.0087 |
| NA12761 | 1.1199 | UC082 | 0.2957 | 1784387 | 0.4779 | DS_13 | 0.0168 |
| NA12762 | 0.9861 | UC083 | 0.6737 | 1784442 | 0.5801 | DS_14 | 0.0845 |
| NA12763 | 0.4475 | UC084 | 0.1378 | 1784515 | 0.3137 | DS_15 | 0.0401 |
| NA12812 | 2.0514 | UC086 | 0.3483 | 1784605 | 0.1372 | DS_16 | 0.0182 |
| NA12813 | 3.2194 | UC087 | 0.7166 | 1784670 | 0.6634 | DS_17 | 0.0132 |
| NA12814 | 1.8483 | UC088 | 0.5847 | 1784692 | 0.0787 | DS_18 | 0.0109 |
| NA12815 | 0.0655 | UC089 | 0.2947 | 1784807 | 3.4925 | DS_19 | 0.0054 |
| NA12872 | 1.7215 | UC090 | 0.1998 | 1784851 | 0.2082 | DS_20 | 0.1502 |
| NA12873 | 0.3562 | UC092 | 0.3562 | 1784950 | 0.0770 | DS_21 | 0.2256 |
| NA12874 | 3.8099 | UC093 | 0.1468 | 1784972 | 0.1672 | DS_22 | 0.1772 |
| NA12875 | 0.0724 | UC094 | 0.2283 | 1785116 | 0.7852 | DS_23 | 0.0616 |
| NA12891 | 0.3399 | UC095 | 0.2888 | 1785209 | 0.0592 | DS_24 | 0.3678 |
| NA12892 | 0.1874 | UC097 | 0.2376 | 1785226 | 0.1782 | DS_25 | 0.1032 |
|  |  | UC098 | 0.4724 | 1785308 | 1.0386 | DS_26 | 0.1555 |
|  |  | UC099 | 1.1626 | 1785319 | 0.6784 | DS_27 | 0.3691 |
|  |  | UC100 | 0.3796 | 1785544 | 0.3467 | DS_28 | 0.2222 |
|  |  | UC103 | 0.6004 | 1785657 | 0.0259 | DS_29 | 0.0368 |
|  |  | UC105 | 0.6383 | 1785668 | 1.2776 | DS_30 | 0.2283 |
|  |  | UC106 | 0.4217 | 1785679 | 0.5872 | DS_31 | 0.0207 |
|  |  | UC108 | 0.2796 | 1785814 | 0.1061 | DS_32 | 0.2268 |
|  |  | UC109 | 0.9818 | 1785888 | 0.2646 | DS_33 | 0.6745 |
|  |  | UC110 | 0.1112 | 1785959 | 0.1349 | DS_34 | 0.4853 |
|  |  | UC112 | 0.2402 | 1786145 | 0.2453 | DS_35 | 1.4539 |
|  |  | UC113 | 0.2400 | 1786163 | 1.2301 | DS_36 | 0.0041 |
|  |  | UC114 | 0.1954 | 1786174 | 1.1839 | DS_37 | 0.0423 |
|  |  | UC115 | 0.9658 | 1786205 | 0.0979 | DS_38 | 0.0141 |
|  |  | UC116 | 0.1841 | 1786292 | 3.7331 | DS_39 | 0.2293 |
|  |  | UC117 | 0.2419 | 1786360 | 0.4318 | DS_40 | 0.2512 |
|  |  | UC119 | 0.3500 | 1786450 | 0.1338 | DS_41 | 0.7611 |
|  |  | UC120 | 0.4724 | 1786532 | 0.3754 | DS_42 | 0.3325 |
|  |  | UC132 | 0.6293 | 1786559 | 0.4289 |  |  |
|  |  | UC135 | 0.5570 | 1786697 | 0.5012 |  |  |
|  |  | UC144 | 0.7143 | 1786867 | 0.1005 |  |  |
|  |  | UC146 | 0.9910 | 1787173 | 0.1513 |  |  |
|  |  | UC149 | 0.5549 | 1787274 | 0.0494 |  |  |
|  |  | UC152 | 0.4312 | 1787566 | 0.4162 |  |  |
|  |  | UC154 | 0.9927 | 1787676 | 0.7196 |  |  |
|  |  | UC155 | 0.5950 | 1787700 | 0.1287 |  |  |
|  |  | UC157 | 0.7993 | 1787744 | 2.6274 |  |  |
|  |  | UC159 | 1.0138 | 1787823 | 0.4910 |  |  |
|  |  | UC160 | 0.8364 | 1787834 | 0.0492 |  |  |
|  |  | UC164 | 0.7476 | 1788262 | 0.4005 |  |  |
|  |  | UC166 | 0.7521 | 1788405 | 0.2647 |  |  |
|  |  | UC167 | 1.8550 | 1788438 | 0.3271 |  |  |
|  |  | UC170 | 0.0500 | 1788677 | 0.2880 |  |  |
|  |  | UC180 | 0.9018 | 1788765 | 0.6170 |  |  |
|  |  | UC182 | 0.3531 | 1788798 | 1.1743 |  |  |
|  |  | UC187 | 0.1103 | 1788833 | 0.0945 |  |  |
|  |  | UC189 | 0.2566 | 1788912 | 0.2451 |  |  |
|  |  | UC190 | 0.2003 | 1789038 | 0.8523 |  |  |
|  |  | UC192 | 0.0795 | 1789049 | 0.1681 |  |  |
|  |  | UC193 | 0.2965 | 1789182 | 0.3005 |  |  |
|  |  | UC194 | 0.0946 | 1789261 | 0.0949 |  |  |
|  |  | UC197 | 0.3289 | 1789417 | 1.7288 |  |  |
|  |  |  |  | 1789613 | 0.4263 |  |  |
|  |  |  |  | 1789689 | 0.3755 |  |  |
|  |  |  |  | 1789900 | 0.0235 |  |  |
|  |  |  |  | 1789958 | 0.3509 |  |  |
|  |  |  |  | 1789975 | 0.2151 |  |  |
|  |  |  |  | 1790065 | 0.2064 |  |  |
|  |  |  |  | 1790098 | 0.3166 |  |  |
|  |  |  |  | 1790114 | 0.3777 |  |  |
|  |  |  |  | 1790177 | 0.1753 |  |  |
|  |  |  |  | 1790213 | 0.2004 |  |  |
|  |  |  |  | 1790295 | 0.0454 |  |  |
|  |  |  |  | 1790323 | 0.0927 |  |  |
|  |  |  |  | 1790334 | 1.0373 |  |  |
|  |  |  |  | 1790340 | 0.3666 |  |  |
|  |  |  |  | 1790444 | 0.0665 |  |  |
|  |  |  |  | 1790475 | 0.5551 |  |  |
|  |  |  |  | 1790497 | 0.1440 |  |  |
|  |  |  |  | 1790608 | 0.5323 |  |  |
|  |  |  |  | 1790709 | 0.1063 |  |  |
|  |  |  |  | 1790726 | 1.3426 |  |  |
|  |  |  |  | 1790737 | 0.0375 |  |  |
|  |  |  |  | 1790945 | 0.0587 |  |  |
|  |  |  |  | 1790980 | 0.1480 |  |  |
|  |  |  |  | 1790991 | 0.4285 |  |  |
|  |  |  |  | 1791220 | 0.4122 |  |  |
|  |  |  |  | 1791242 | 0.2151 |  |  |
|  |  |  |  | 1791288 | 1.6206 |  |  |
|  |  |  |  | 1791367 | 0.3501 |  |  |
|  |  |  |  | 1791423 | 0.1577 |  |  |
|  |  |  |  | 1791591 | 0.0822 |  |  |
|  |  |  |  | 1791607 | 2.4070 |  |  |
|  |  |  |  | 1791809 | 0.4557 |  |  |
|  |  |  |  | 1791826 | 1.3596 |  |  |
|  |  |  |  | 1791848 | 0.1123 |  |  |
|  |  |  |  | 1791860 | 0.5543 |  |  |
|  |  |  |  | 1791949 | 0.3534 |  |  |
|  |  |  |  | 1792012 | 0.8552 |  |  |
|  |  |  |  | 1792175 | 0.2814 |  |  |
|  |  |  |  | 1792314 | 0.4888 |  |  |
|  |  |  |  | 1792377 | 0.4017 |  |  |
|  |  |  |  | 1792402 | 0.0832 |  |  |
|  |  |  |  | 1792489 | 0.5513 |  |  |
|  |  |  |  | 1792564 | 0.4437 |  |  |
|  |  |  |  | 1792628 | 0.0980 |  |  |
|  |  |  |  | 1792691 | 0.0544 |  |  |
|  |  |  |  | 1792707 | 0.3488 |  |  |
|  |  |  |  | 1792872 | 0.2429 |  |  |
|  |  |  |  | 1792894 | 0.9924 |  |  |
|  |  |  |  | 1793134 | 0.2295 |  |  |
|  |  |  |  | 1793185 | 0.2274 |  |  |
|  |  |  |  | 1793258 | 1.5591 |  |  |
|  |  |  |  | 1793376 | 0.2295 |  |  |
|  |  |  |  | 1793431 | 0.2706 |  |  |
|  |  |  |  | 1793571 | 0.0867 |  |  |
|  |  |  |  | 1793670 | 0.2791 |  |  |
|  |  |  |  | 1793846 | 0.1007 |  |  |
|  |  |  |  | 1793873 | 1.4094 |  |  |
|  |  |  |  | 1793908 | 1.0845 |  |  |
|  |  |  |  | 1793936 | 0.3851 |  |  |
|  |  |  |  | 1794113 | 0.0954 |  |  |
|  |  |  |  | 1794223 | 0.2267 |  |  |
|  |  |  |  | 1794257 | 0.4686 |  |  |
|  |  |  |  | 1794358 | 0.0770 |  |  |
|  |  |  |  | 1794476 | 2.0866 |  |  |
|  |  |  |  | 1794508 | 0.4946 |  |  |
|  |  |  |  | 1794572 | 0.5536 |  |  |
|  |  |  |  | 1794648 | 0.0753 |  |  |
|  |  |  |  | 1794654 | 0.8998 |  |  |
|  |  |  |  | 1794738 | 0.2897 |  |  |
|  |  |  |  | 1794770 | 0.4031 |  |  |
|  |  |  |  | 1794845 | 0.4599 |  |  |
|  |  |  |  | 1794907 | 1.1831 |  |  |
|  |  |  |  | 1794951 | 0.1378 |  |  |
|  |  |  |  | 1795005 | 0.0787 |  |  |
|  |  |  |  | 1795109 | 0.4071 |  |  |
|  |  |  |  | 1795126 | 0.5454 |  |  |
|  |  |  |  | 1795171 | 0.2571 |  |  |
|  |  |  |  | 1795261 | 0.0597 |  |  |
|  |  |  |  | 1795307 | 0.0982 |  |  |
|  |  |  |  | 1795335 | 0.0791 |  |  |
|  |  |  |  | 1795346 | 0.0258 |  |  |
|  |  |  |  | 1795384 | 0.0608 |  |  |
|  |  |  |  | 1795439 | 0.1998 |  |  |
|  |  |  |  | 1795512 | 0.1830 |  |  |
|  |  |  |  | 1795534 | 0.5294 |  |  |
|  |  |  |  | 1795579 | 0.1470 |  |  |
|  |  |  |  | 1795596 | 0.2816 |  |  |
|  |  |  |  | 1795624 | 0.3712 |  |  |
|  |  |  |  | 1795859 | 0.5024 |  |  |
|  |  |  |  | 1796045 | 0.1574 |  |  |
|  |  |  |  | 1796080 | 2.1635 |  |  |
|  |  |  |  | 1796116 | 0.4967 |  |  |
|  |  |  |  | 1796127 | 1.1144 |  |  |
|  |  |  |  | 1796319 | 0.8839 |  |  |
|  |  |  |  | 1796347 | 0.2486 |  |  |
|  |  |  |  | 1796435 | 0.3028 |  |  |
|  |  |  |  | 1796533 | 0.2504 |  |  |
|  |  |  |  | 1796640 | 0.2564 |  |  |
|  |  |  |  | 1796702 | 0.2270 |  |  |
|  |  |  |  | 1796778 | 0.2398 |  |  |
|  |  |  |  | 1796899 | 0.3547 |  |  |

**Supplementary Table S1: Sample collection identifiers and phenotype**

All individuals used in this study are indicated with their identifier and their phenotype. H_2_O_2_ release indicates normalized hydrogen peroxide release levels, units are arbitrary.
