# Supplementary figures and images for: Extensive Natural Variation for Cellular Hydrogen Peroxide Release Is Genetically Controlled
Source: PLoS One. 2012 Aug 29;7(8):e43566. doi: 10.1371/journal.pone.0043566 (PMC3430705; doi:10.1371/journal.pone.0043566)

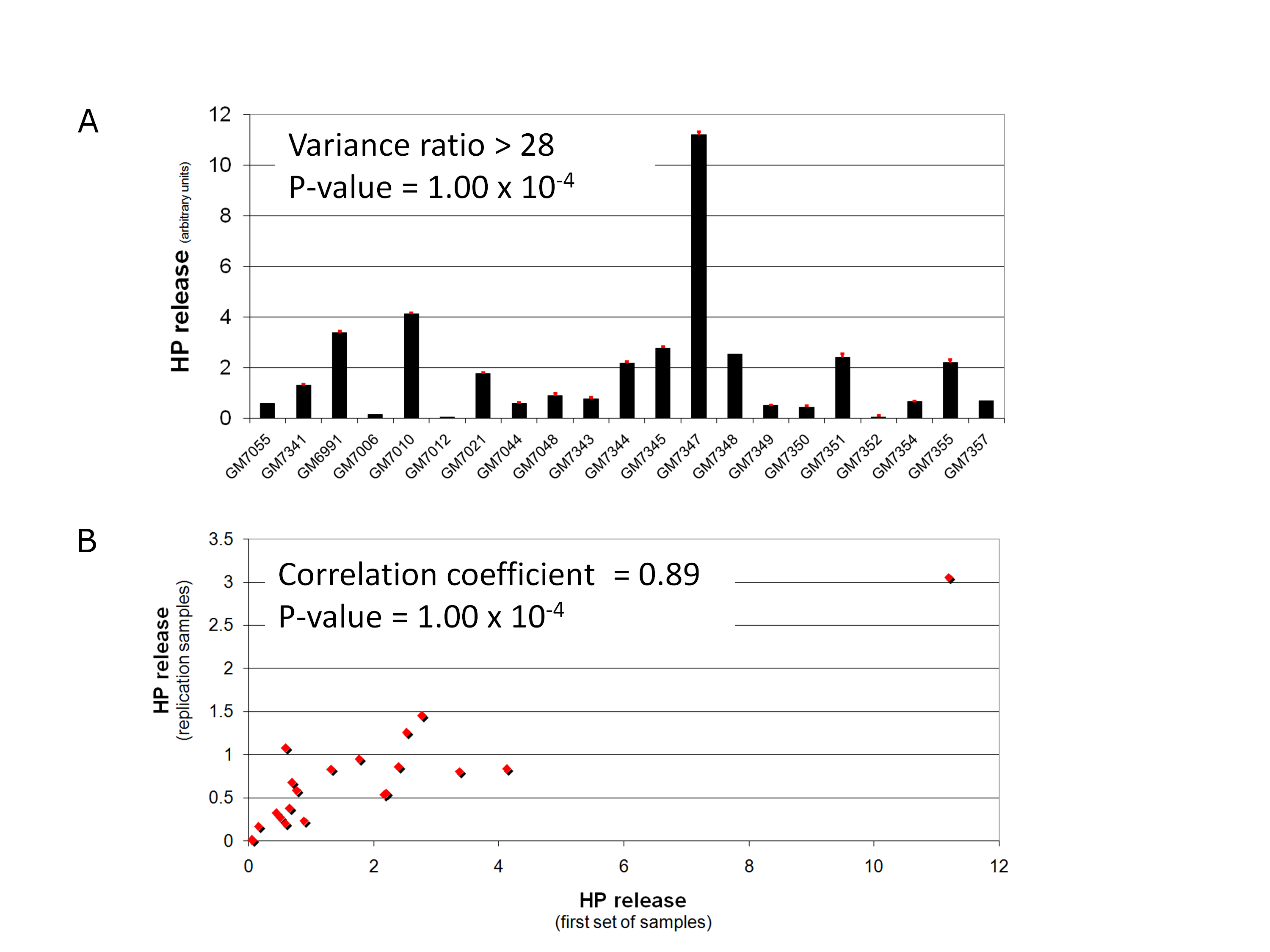

Supplement: File S2 — Extensive inter-individual variation and high reproducibility for H2O2 phenotypes. (A) H2O2 release for 21 CEPH-HapMap unrelated individuals, each of which was measured in 4 replicates is shown. Each bar corresponds to the normalized H2O2 release of an individual, and standard deviations of three replicates per individual are indicated in red. The coefficient of variance is >28 with a p-value of p<1.00×10^−4^, indicating that there is 28 times more variation among individuals than among replicates. (B) H2O2 release for the same 21 individuals was measured several weeks later, to estimate the reproducibility of the phenotype. Comparison for individuals of both experimental sets indicates a coefficient of correlation of more than 0.89 (Pearson correlation test, p<1.00×10^−4^). (TIF) [file pone.0043566.s002.tif]

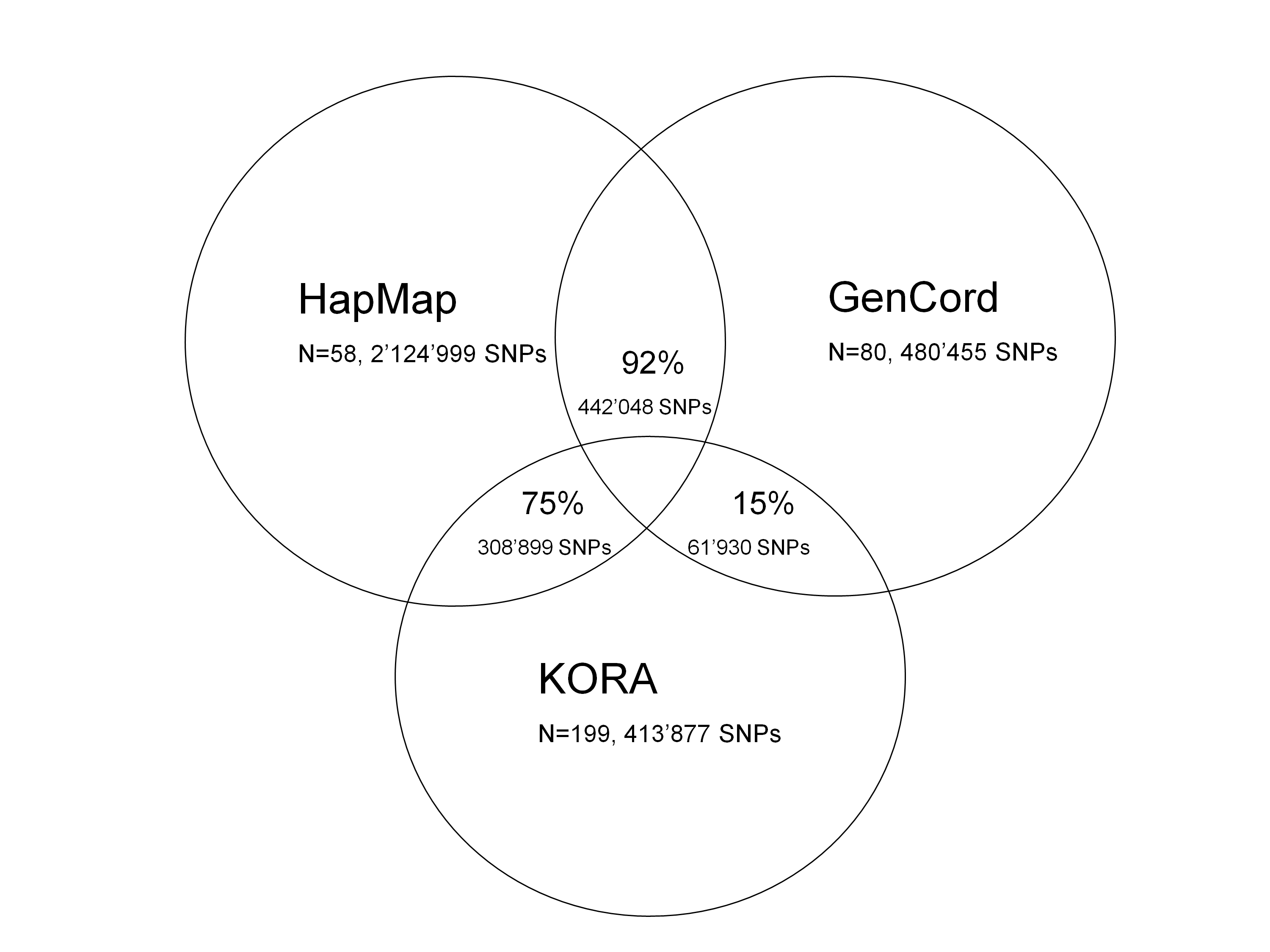

Supplement: File S3 — Overlap of SNPs among the three sample cohorts and genotyping platforms. This schematic figure displays the number of individuals genotyped for each cohort, and the number of SNPs genotyped per population. The overlap between the circles shows the numbers and percentages of overlapping SNPs among populations and genotyping platforms. (TIF) [file pone.0043566.s003.tif]

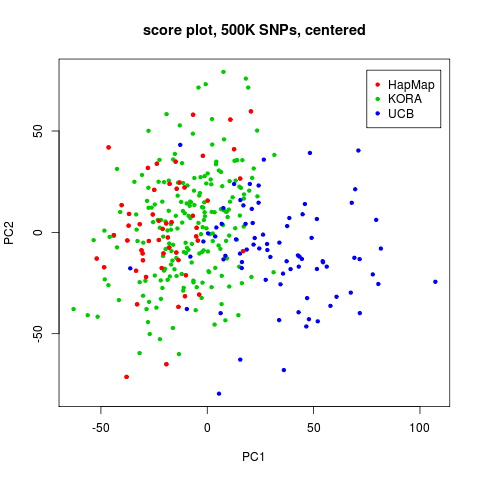

Supplement: File S4 — Principal component analysis indicates no apparent substructure. A random set of 500,000 SNPs of 337 individuals from three cohorts was analyzed using centered principal component analysis (PCA). Here we show PC1 versus PC2. No evident substructure is apparent among cohorts. (PNG) [file pone.0043566.s004.png]
